# Prediction of Fragility Fractures and Mortality in a Cohort of Geriatric Patients

**DOI:** 10.1002/jcsm.13631

**Published:** 2024-11-08

**Authors:** Peter Dovjak, Bernhard Iglseder, Anna Rainer, Gregor Dovjak, Michael Weber, Peter Pietschmann

**Affiliations:** ^1^ Department of Acute Geriatrics Salzkammergut Clinic Gmunden Gmunden Austria; ^2^ Department of Geriatric Medicine, Christian Doppler Hospital Paracelsus Medical University Salzburg Austria; ^3^ Department of Biomedical Imaging and Image‐Guided Therapy Medical University of Vienna Vienna Austria; ^4^ Institute of Pathophysiology and Allergy Research, Center for Pathophysiology, Infectiology and Immunology Medical University of Vienna Vienna Austria

**Keywords:** fragility fracture, geriatric patient, osteosarcopenia, sarcopenic obesity

## Abstract

**Background:**

Risk factors of refracture after fragility fractures include osteoporosis, female gender and advanced age among others. We hypothesized that the assessment of functionality, muscle health and nutrition status contribute to the risk prediction for further fractures and death.

**Methods:**

We assessed 334 patients admitted to the department of acute geriatrics for sociodemographic data, bone fragility, selected laboratory tests, body composition and data on functionality using the comprehensive geriatric assessment. Patients had follow‐ups until the occurrence of further fractures or death. Dual‐energy X‐ray absorptiometry and pulse echo measurements were performed to assess bone mineral density. Fracture risk was assessed using the FRAX score and muscle strength according to published guidelines on sarcopenia.

**Results:**

The mean age was 81 years (70–95), and 82.3% (275/334) were women. An incidence of 10.4% (35/334) new fragility fractures was observed within 24 months, and the mortality rate was 12.2% (41/334). A significantly higher rate of further fractures was associated with lower BMI (body mass index) (HR 0.925, CI 0.872–0.98; *p* = 0.009), lower parathyroid hormone levels (HR 0.986, CI 0.973–0.998; *p* = 0.026) and with the diagnosis of osteoporosis (HR 2.546, CI 1.192–5.438; *p* = 0.016). No significant associations were present in patients with previous fractures, with higher age, higher FRAX scores, sarcopenia, in women, sarcopenic obesity, frail patients, lower grip strength, lower walking speed, lower Barthel index or lower DI (density index) values. The predictive power for further fractures was 10.7% higher adding osteosarcopenia, BMI and parathyroid hormone levels to standard assessment parameters osteoporosis, age and the status of previous fractures. Mortality was significantly higher with advanced age (HR 1.101, CI 1.052–1.151; *p* < 0.001), in men (HR 6.464, CI 3.141–13.305; p < 0.001), in smokers (*p* = 0.002), higher FRAX score (HR 1.039, CI 1.009–1.070; *p* = 0.010), lower renal function (HR 0.987, CI 0.976–0.997; p = 0.010), lower Tinetti test scores (HR 0.943, CI 0.900–0.987; *p* = 0.012), lower walking speed (HR 0.084, CI 0.018–0.382; *p* = 0.001), lower hand grip (HR 0.876, CI 0.836–0.919; *p* < 0.001) and lower Barthel index scores (HR 0.984, CI 0.971–0.997; *p* = 0.015).

**Conclusions:**

In a cohort of geriatric patients, the addition of BMI, low parathyroid hormone levels and osteosarcopenia increases the predictive power for further fractures by 10.7%. These parameters are a valuable addition to the standard assessment parameters age and history of sustained fractures. Mortality is partly associated with potentially treatable functional parameters.

## 1 Introduction

Fragility fractures, defined as fractures caused by a fall from standing height or less, are most commonly induced by osteoporosis [1]. The sequelae of hip fractures, the most feared fragility fracture, are dramatic: Only 40%–60% of hip fracture survivors are likely to recover their pre‐fracture level of mobility, while 40%–70% of people may regain their pre‐fracture level of independence for composite measures in daily living [2]. The prevalence of these fractures differs between men and women and increases with age [3].

Longevity increases the proportion of older people worldwide, a new challenge in the medical care of the age‐associated diseases such as osteoporosis, sarcopenia, frailty and other well‐known geriatric syndromes [4]. In 1950, no country worldwide had more than 11% of its population over the age of 65, and in 2000, the highest rate was 18%. But in 2050, projections indicate that there will be a rate of 38% of older people [5]. The ageing process is heterogeneous, so chronologic age alone is a poor descriptor of fracture risk. A person over 70 years of age is defined as geriatric if they show typical multimorbidity (geriatric syndromes are present) or if they are already over 80 years of age [6]. Frailty affects 15% of older adults in the United States, and 45% of whom are prefrail [7]. Physical frailty was originally constructed in the cardiovascular health study including five questions about unintentional weight loss, weakness or poor handgrip strength, self‐reported exhaustion, slow walking speed and low physical activity.

The diagnosis of osteoporosis is based on a low bone mineral density (BMD) quantified with dual‐energy X‐ray absorptiometry (DXA). A treatment gap related to a low diagnostic rate was above 50% in 8 countries in Europe. For older patients with functional decline, a screening test for osteoporosis and/or fracture risk at the point of care could bridge the diagnostic gap providing a more convenient diagnosis [8]. An algorithm was proposed in 2008; the World Health Organization developed a fracture risk assessment tool named FRAX to determine the risk of fracture and created a web‐based program for different countries [3]. Based on an algorithm using FRAX and pulse‐echo ultrasound data, a clinical decision regarding specific treatment for osteoporosis could be made in 90.9% in older patients in a easily accessible and convenient point of care setting in a cohort of 333 geriatric patients [9].

Many fragility fractures occur in patients with normal bone density values or osteopenia on DXA, emphasizing the need for better detection of fracture risk [10]. Osteoporosis in men is underdiagnosed, in both primary and secondary preventions of fragility fractures [11].

Published risk factors of refracture include osteoporosis, female gender and older age at time of the first fracture [1]. However, in an Australian study over the period of 20 years, 65% of all fractures were not caused by advancing age and osteoporosis [10].

A novel risk factor for fragility fractures is sarcopenia as part of the frailty syndrome [12]. Reduced muscle mass, which follows reduced muscle strength, enhances balance problems and hampers the adaptive bone remodelling [13]. Patients with sarcopenia had a 60% higher risk of falls and an 84% higher risk of fractures as non‐sarcopenic patients due to a meta‐analysis by Yeung et al. [14]. Sarcopenia was more prevalent in older men after a fragility fracture than in women. Sarcopenia and the combination with osteoporosis—osteosarcopenia—can be accurately diagnosed using the algorithm proposed by national working groups on sarcopenia [15]. Both entities are associated with both higher mortality and refracture rate [16]. The term osteosarcopenia was titled as geriatric giant of the 21st century by Duque et al. and describes patients with low muscle mass and low muscle function in addition to osteoporosis [17]. In overweight persons, sarcopenia is often overseen but highlights a relevant clinical syndrome called sarcopenic obesity and is associated with a higher rate of falls, lower walking speed and frailty [18].

Global demographic changes are associated with a higher prevalence of osteoporosis and fragility fractures. In order to develop preventive strategies, we need improved risk management and a better identification of preventable risk factors. Therefore, we designed a study in geriatric patients to analyse muscle, bone and functionality parameters in addition to routine clinical parameters to predict further fractures and mortality. We hypothesized that the parameters of the functionality assessment, muscle health and nutrition status contribute to the risk prediction for further fractures and death.

## 2 Methods

### 2.1 Study Population

From September 2018 to July 2021, 3448 patients were admitted to the department of acute geriatrics. Based on the inclusion criteria, we recruited 334 patients for the study. Due to the large number of patients with functional decline (Table 2), only 9.7% of the admitted patients agreed to participate in the study. The sample size was calculated for the underlying cohort to study the accuracy of the pulse‐echo ultrasound measurement for the detection of osteoporosis on an anticipated dropout rate of 10%. The probability of a type‐I‐error was set to 0.05. A minimum of 335 patients had to be recruited to determine a power of effect of 0.8 [9]. This cohort, aged 70–95 years, was assessed for sociodemographic data, parameters of bone fragility, body composition and data on functionality using the comprehensive geriatric assessment. We followed these patients prospectively for the occurrence of further fractures and death. In this study, we present the data of the follow up in the first 24 months.

### 2.2 Measurement at Baseline

#### 2.2.1 Participants

All men and women with risk factors according to the guidelines for osteoporosis admitted to the department were reviewed by the study coordinator (P.D.) [19]. Patients with active treatment for osteoporosis, those with amputated lower extremities, with a neoplastic or inflammatory bone disease or patients unable to provide written consent were excluded (details in Supporting Information S1). Participants provided their informed approval on a declaration of consent form. A geriatrician took medical history and the physical examination. Functional abilities were tested using tools of the comprehensive geriatric assessment during an obligatory examination of all patients admitted to the department of acute geriatrics [20]. Diagnosis of a geriatric patient was based on the definition of the German Society for Geriatrics and Gerontology [6]. Fragility fracture was defined as a fracture that occurred spontaneously or followed a minor trauma, such as a fall from standing height or a height less than a meter, a fall from sitting or a fall from laying down from less than a meter high, a fall after having missed one to three steps, after a movement outside of the typical plane of motion or coughing [21]. Laboratory tests were obligatory for all admissions as part of the clinical routine, and DXA, fracture risk assessment and muscle mass measurement were performed as indicated in accordance the guidelines for osteoporosis and sarcopenia [15].

#### 2.2.2 Dual X‐Ray (DXA) and Pulse Echo Measurements

Using the Lunar Prodigy densitometer (General Electric Company, Madison, WI, USA) in the Department of Radiology, BMD was measured at the femoral neck and at the lumbar spine. Radiation dosage was < 0.1 μGy. Quality control was performed daily using a specific software provided by the company. Both hips were scanned in patients without joint replacements or osteosynthetic material from previous orthopaedic surgery. The lowest T‐scores were analysed in the study. Osteoporosis was diagnosed on a T‐score ≤ − 2.5 in accordance with the WHO definition [22].

A point‐of‐care instrument provided by Bone Index Finland, Kuopio, Finland (Bindex), was used for pulse‐echo ultrasound measurement. The Probe was connected to a universal serial bus port of a personal computer. In this device, an electrical pulse is transmitted into a 3.0‐MHz ultrasound wave that was sent through the skin of the proximal tibia. The exact site of measurement was found using anatomical landmarks based on the manufacturer's rules. After applying ultrasound gel, the transducer was moved over the tibia, repeating the manoeuvre five times. The mean value of all measurements was obtained for the analysis. The same observer (A.R.) provided all data. Multiplying the time lag between the ultrasound echoes from the periosteal to the endosteal surface was calculated by the software of the device resulted the cortical thickness. Combined with data regarding patient's age, gender, weight, and height we assessed values of the cortical thickness. These values were calculated by an algorithm and expressed as DI, an estimation of BMD [23].

#### 2.2.3 FRAX Score

Fracture risk was calculated using the FRAX score based on patients data including age, gender, weight, height and several other risk factors (prior fragility fracture, parental history of hip fractures, current smoking habits, current use of long‐term glucocorticoids, rheumatoid arthritis, other causes of secondary osteoporosis and alcohol consumption) calibrated to the known epidemiologic situation in Austria [24]. The data were calculated without measuring BMD to avoid collinearity with the values of the DI and DXA measurements.

#### 2.2.4 Anthropometric and Muscle Mass Measurements

Weight was measured in all patients using a calibrated seat scale Type 7702 (Soehnle company, Germany) wearing hospital clothing. Height information was collected according to reported information or tape measurement in case of plausibility doubts. Weight and height values resulted in the BMI using the formula: weight (kg) divided by height squared (m^2^). Appendicular skeletal muscle mass (ASM) was calculated using the densitometer described above. The sum of the lean mass values (in kg) of legs and arms divided by the squared body height resulted in the skeletal mass‐index (SMI) in accordance to published formula [25]. The cut‐off values proposed by the European working group on sarcopenia were used for the diagnosis of sarcopenia [26]. Osteosarcopenia was diagnosed in patients with osteoporosis and sarcopenia using the algorithms proposed by Kirk et al. [26]. Sarcopenic obesity were counted in patients along the consensus statement of the European Society for Clinical nutrition and Metabolism [27].

#### 2.2.5 Outcome Assessment

All participants of the study were followed until 23 April 2023 using the electronic patients' files of the upper Austrian health care holding company. Diagnosis and events were confirmed by the attending physician and the World Health Organization's International Classification of Diseases 10th Revision (ICD‐10: S00‐S99 and M80‐M82). The public death register comparison was carried out to calculate the mortality rate.

#### 2.2.6 Statistical Analysis

All statistical computations were performed using IBM SPSS Statistics for Windows version 28 (IBM, Armonk, NY). Metric data are described using mean ± standard deviation (SD) if approximately normally distributed or median [min, max] in case of skewed data. Nominal data are described using absolute frequencies and percentages. Unpaired Student's *t*‐test (in case of normally distributed metric data), Mann–Whitney *U* test (given ordinal or skewed metric data) or chi^2^ test (for nominal data) were used to compare as well patients who died to patients who survived as patients with and without new fractures within 24 months. In order to model the impact of variables on mortality or fracture, multiple binary logistic regressions were used. In these models, only predictors were used that differed significantly between groups. Additionally, cox regressions were used to model the impact of various predictors on time to mortality or time to fracture, respectively. A *p* value equal or below 0.05 was considered statistically significant. In order to avoid an increased error of the second type, no multiplicity corrections were performed.

## 3 Results

From September 2018 to July 2021, 334 patients were recruited for the study. The average age was 81 years (70–95), and 82.3% (275/334) were women, equivalent to the age‐adjusted sex distribution in the Austrian population. The presence of fragility fracture already present at baseline (58%) raised the concern about a sequent fragility fracture (‘fracture cascade’) in the follow‐up period [28] (Table 1). The rate of patients with frailty (79.1%), sarcopenia (20.7%) and low Barthel index (49.7%) reflected the high number of geriatric syndromes in our cohort (Tables 1 and 2).

**TABLE 1:** Characteristics of the study population stratified by occurrence of new fractures and survival within 24 months.

Characteristics	Total (334)	No further fracture (299)	New fracture (35)	*p*	Survived (293)	Deceased (41)	*p*
Mean age in years; (min/max)	81 (70/95)	81 (70/95)	79 (70/89)	0.351	80 (70/94)	84 (72/95)	**0.004**
Women, number (%)	275 (82.3)	247 (82.6)	28 (80)	0.702	243 (82.9)	32 (78)	0.476
Men, number (%)	59 (17.7)	52 (17.4)	7 (20)		50 (17.1)	9 (22)	
Body mass index (kg/m^2^); (min/max)	26 (15/45.3)	25.5 (15/45.3)	23.8 (17.8/34.4)	**0.027**	25.5 (16.9/45.3)	23.8 (15/41.6)	**0.008**
Glomerular filtration rate (mL/min); (min/max)^a^	74 (19/166)	72 (19/166)	73 (21/107)	0.789	74 (21/166)	61 (19/125)	**0.014**
Appendicular muscle mass index (kg/m^2^)	6.1 (3.5/13.5)	6.2 (3.5/13.5)	5.7 (4.7/8.9)	0.209	6.09 (3.5/12.5)	6.05 (4.02/9.89)	0.856
Low appendicular muscle mass index (%)^b^	111 (33.5)	95 (32)	16 (47.1)	0.105	94 (32.2)	17 (43.6)	0.171
Sarcopenia, number (%)^b^	69 (20.7)	60 (20.1)	9 (25.7)	0.280	58 (19.8)	11 (26.8)	0.169
Obese sarcopenia^c^	4 (1.2)	4 (1.3)	0	0.641	4 (1.4)	0	0.453
Dual X‐ray absorptiometry, T‐value (min/max)	−1.7 (−5.6/6.1)	−1.7 (−5.6/6.1)	−2.3 (−4.9/2.9)	**0.021**	−1.8 (−5.6/6.1)	−1.7 (−4.3/1.6)	0.508
Osteoporosis, number (%)^d^	89 (26.7)	74 (24.8)	15 (42.9)	**0.017**	75 (25.9)	14 (35)	0.165
Density index (min/max)	0.79 (0.61/1.27)	0.77 (0.61/1.,27)	0.77 (0.64/0.95)	0.350	0.78 (0.63/1.27)	0.74 (0.611/0.96)	**0.035**
FRAX score (%), without bone mass measurement, risk for major fractures (min/max)	26.7 (6/51)	27 (6.1/51)	29 (7.2/42)	0.792	27 (6.1/51)	27.5 (9.8/42)	0.329
Parathyroid hormone	43 (8/376)	45 (8/376)	36 (18/103)	0.067	43 (8/275)	47.5 (8/376)	0.444
Fractures at admission (%)	197 (58.9)	173 (57.9)	24 (68.6)	0.223	171 (58.4)	26 (63.4)	0.493

*Note:* Data with significant values are presented in bold.

^a^ Estimate using the MDRD formula (Modification of Diet in Renal Disease Study).

^b^ Definition and cut off values according to the revised European consensus 2019 by Cruz‐Jentoft et al.

^c^ Obese sarcopenia: BMI ≥ 30 in sarcopenic patients.

^d^ Based on the WHO Definition on the diagnosis of osteoporosis.

**TABLE 2:** Functionality assessment of the study population stratified by occurrence of new factures and survival within 24 months.

Characteristics	Total (334)	No further fractures (299)	New fractures (35)	*p*	Survived (293)	Deceased (41)	*p*
Barthel index, mean; (min/max)		80 (20/100)	85 (25/95)	0.893	85 (20/100)	80 (35/100)	0.084
Barthel index below 80 points, number (%)	166 (49.7)	150 (50.2)	16 (45.7)	0.231	144 (49.1)	22 (53.7)	**0.024**
Handgrip strength (kg) men, (min/max)	27.8 (14.6/47.3)	26 (14.6/47.3)	29 (16.6/42.6)	0.626	26 (14.6/47.3)	28 (16.6/35.3)	0.509
Handgrip strength (kg) women, (min/max)	16.5 (4/35.6)	16.6 (4/35.6)	15.3 (6/25.6)	0.174	17 (4/35.6)	13.2 (5/20)	**< 0.001**
Low handgrip strength, number (%)	163 (49.8)	143 (48.6)	20 (60.6)	0.193	135 (47.2)	28 (68.3)	**0.011**
Walking speed (m/s)	0.5 (0.1–1.0)	0.5 (0.1/1)	0.5 (0.3/1)	0.956	0.5 (0.3/1)	0.5 (0.1/0.8)	**0.002**
Weight loss (311), number (%)	90 (28.9)	83 (30.1)	7 (20)	0.216	77 (28.1)	13 (35.1)	0.375
Stable weight	221 (71.1)	193 (69.9)	28 (80)		197 (71.9)	24 (64.9)	
Frail patients, number (%)^a^	246 (79.1)	220 (79.4)	26 (76.5)	0.690	211 (77.6)	35 (89.7)	0.080
Prefrail or non‐frail	65 (20.9)	57 (20.6)	8 (23.5)		61 (22.4)	4 (10.3)	

*Note:* Data with significant values are presented in bold.

^a^ Frailty was calculated on low grip strength (< 26 kg in men and < 15 kg in women), low walking speed (< 0.8 m/s), low Barthel index (≤ 80 points) and weight loss in last 3 months; frail patients had 2 or more points positive.

Within 24 months, a cumulative incidence of 10.4% new fragility fractures were identified, a lower rate as in a retrospective analysis of 11 833 Swedish female patients in a similar follow up time (16.3%). A cross‐tabulation with univariate analysis of risk factors for further fractures according to the literature was performed [29]. A significantly higher rate of further fractures was associated with lower BMI and with the diagnosis of osteoporosis, respectively, lower T‐values. No significant associations were present in patients with previous fractures, with higher age, higher FRAX scores, with sarcopenia, in women, frail patients, lower handgrip, lower walking speed, lower Barthel index or lower DI values (Tables 1 and 2). Despite the published data on a higher risk for falls (and subsequent fractures), we could not find a significant association between sarcopenic obesity and further fractures in our cohort. We did a crosstab analysis of comorbidity and splitted our cohort to age groups that revealed no other significant risk factors for fractures. Mortality data on age groups confirmed the trend presented in Table 1. Mortality was significantly higher in smokers (*p* = 0.002), patients on glucocorticoid medication (*p* = 0.043), with chronic obstructive respiratory disease (*p* = 0.006) and malnutrition (*p* = 0.006) that goes in line with the trend shown with lower BMI and lower grip strength in Table S1.

Because of the overlaps of patients diagnosed with osteoporosis, who were also affected with sarcopenia and vice versa, we made a cross tabulation, isolating each factor. We analysed patients with isolated osteoporosis and sarcopenia alone together with patients with osteosarcopenia or none and the association with further fractures and death. This analysis yielded significant data only in women (*p* = 0.023), but neither in men nor in the total cohort (Table 3).

**TABLE 3:** Cross tabulation of new fractures and mortality within 24 months in association with isolated osteoporosis, sarcopenia and osteosarcopenia in total (A), women (B) and men (C).

(A) Total							
Parameters	Total (334)	No further fracture (298)	New fracture (35)	*p*	Survived (293)	Deceased (41)	*p*
Osteoporosis (−), sarcopenia (−)	204	186 (91.6)	17 (8.4)	0.087	183 (90.1)	20 (9.9)	0.430
Osteoporosis (+), sarcopenia (−)	57	49 (86)	8 (14)		49(86)	8 (14)	
Sarcopenia (+), osteoporosis (−)	41	38 (92.7)	3 (7.3)		35 (85.4)	6 (14.6)	
Osteosarcopenia	32	25 (78.1)	7 (21.9)		26 (81.3)	6 (18.8)	

(B) Women							
Parameters	Total (275)	No further fractures (247)	New fracture (28)	*p*	Survived (243)	Deceased (32)	*p*
Osteoporosis (−), sarcopenia (−)	170	157 (92.4)	13 (7.6)	**0.023**	153 (90)	17 (10)	0.603
Osteoporosis (+), sarcopenia (−)	52	46 (88.5)	6 (11.5)		45 (86.5)	7 (13.5)	
Sarcopenia (+), osteoprosis (−)	27	25 (92.6)	2 (7.4)		45 (86.5)	5 (13.5)	
Osteosarcopenia	26	19 (73.1)	7 (26.9)		23 (88.5)	3 (11–5)	

(C) Men							
Parameters	Total (59)	No further fractures (52)	New fracture (7)	*p*	Survived	Deceased	*p*
Osteoporosis (−), sarcopenia (−)	34	30 (88.2)	4 (11.8)	0.180	30 (88.2)	4 (11.8)	0.081
Osteoporosis (+), sarcopenia (−)	5	3 (60)	2 (40)		4 (80)	1 (20)	
Sarcopenia (+), osteoprosis (−)	14	13 (42.9)	1 (7.1)		12 (42.9)	1 (7.3)	
Osteosarcopenia	6	6 (100)	0 (0)		3 (50)	3 (50)	

*Note:* Data with significant values are presented in bold.

The cox regression analysis of parameters at baseline and time to events was performed to determine the influence of the relevant variables described in the literature on the time without further fracture and the survival time. We found a significant association of a lower BMI, osteoporosis and lower parathyroid hormone levels with the risk of further fragility fractures. Additionally, a lower ASM index was significant predictor for further fractures (Table 4).

**TABLE 4:** Cox regression of time to fracture and time to death in association with patient's characteristics at baseline.

(A) Hazard ratio of parameters at baseline and time to fracture				
Parameter	Hazard ratio	Lower bound	Upper bound	*p*
Age	1.001	0.956	1.048	0.970
Gender	1.345	0.578	3.129	0.491
Glomerular filtration rate	1.000	0.990	1.010	0.999
ASM index	0.728	0.564	0.942	**0.016**
Body mass index	0.925	0.872	0.980	**0.009**
Total protein level	1.000	0.961	1.041	0.991
25‐Hyroxy vitamin D level	0.998	0.981	1.016	0.845
Parathyroid hormone level	0.986	0.973	0.998	**0.026**
Osteoporosis	2.546	1.192	5.438	**0.016**
Density index	0.211	0.012	3.712	0.288
FRAX score—risk for hip fracture	1.030	0.999	1.062	0.062
Tinetti test	0.970	0.924	1.019	0.229
Walking speed	0.947	0.234	3.838	0.939
Hand grip	0.961	0.918	1.007	0.092
Barthel index	0.998	0.983	1.014	0.835

(B) Hazard ratio of parameters at baseline and time to death				
Parameter	Hazard ratio	Lower bound	Upper bound	*p*
Age	1.101	1.052	1.151	**< 0.001**
Gender	6.464	3.141	13.305	**< 0.001**
Glomerular filtration rate	0.987	0.976	0.997	**0.010**
ASM index	0.939	0.745	1.184	0.597
Body mass index	0.947	0.895	1.001	0.053
Total protein level	1.021	0.987	1.056	0.223
25‐Hydroxy vitamin D level	0.987	0.969	1.006	0.175
Parathyroid hormone level	1.004	0.998	1.010	0.177
Osteoporosis	0.896	0.484	1.657	0.725
Density index	0.080	0.003	1.489	0.089
FRAX score—risk for hip fracture	1.039	1.009	1.070	**0.010**
Tinetti test	0.943	0.900	0.987	**0.012**
Walking speed	0.084	0.018	0.382	**0.001**
Hand grip	0.876	0.836	0.919	**< 0.001**
Barthel index	0.984	0.971	0.997	**0.015**

*Note:* Data with significant values are presented in bold.

**FIGURE 1:**
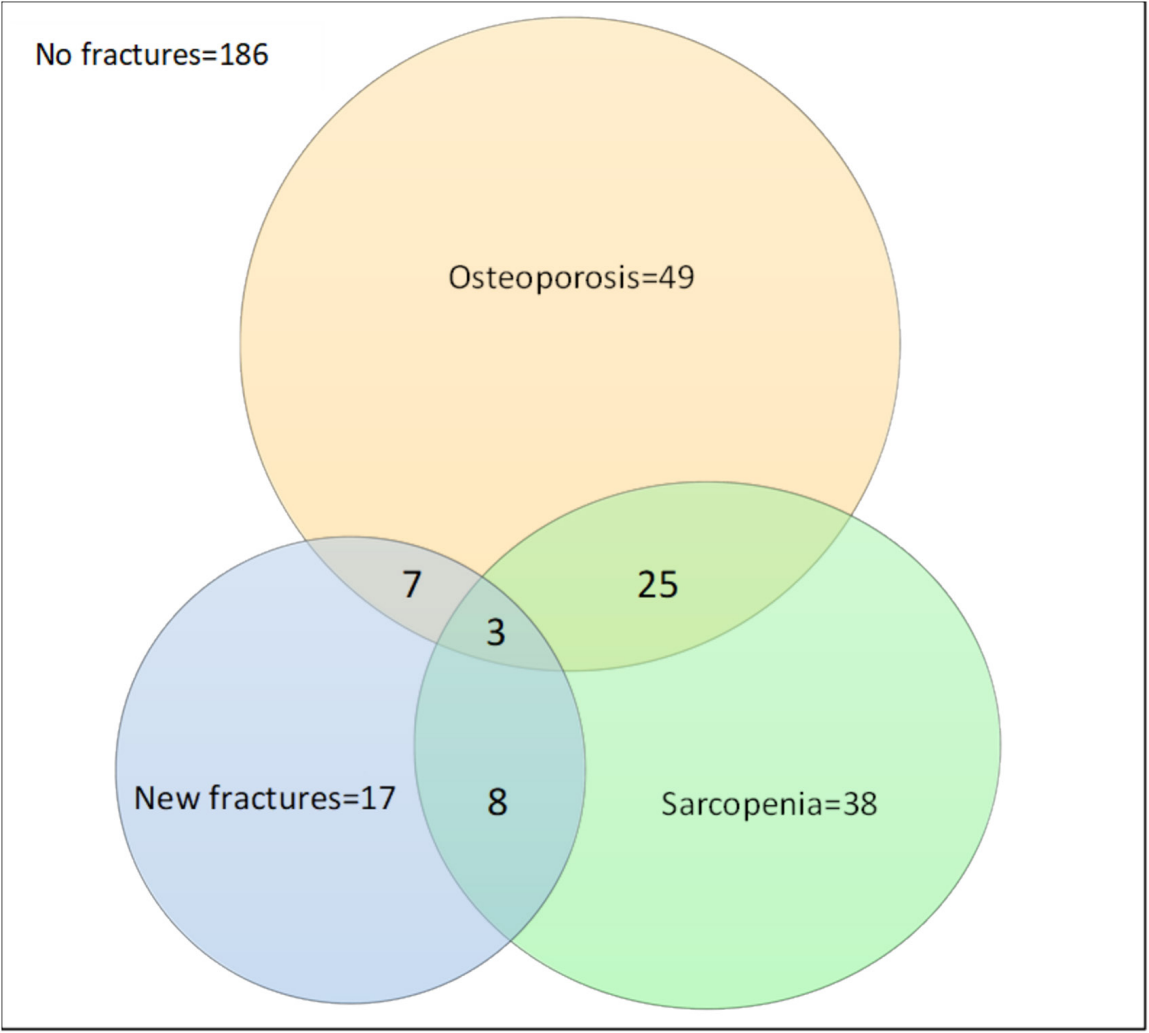
Venn diagram of patients with new fractures within 24 months.

The Venn diagram was constructed to visualize the intersections of osteoporosis, sarcopenia and osteosarcopenia with subsequent fractures. In this diagram, an overlap with osteoporosis in 47% of new fractures, 17.6% with sarcopenia and 41.2% with both entities was demonstrated, specifying the results of the presented analysis (Figure 1).

The mortality analysis using the cross tabulation revealed a significantly higher rate in patients with advanced age (*p* = 0.004), with lower BMI (*p* = 0.008), lower glomerular filtration rate (*p* = 0.014), lower DI‐value (*p* = 0.035), lower Barthel index (*p* = 0.024), lower hand grip strength (*p* = 0.011), in smokers (*p* = 0.002), patients on long term corticoid medication (*p* = 0.043), patients with chronic obstructive respiratory disease (*p* = 0.006), malnutrition (*p* = 0.006) and slower walking speed (*p* = 0.002). Survival rate was not influenced by gender, bone parameters, sarcopenia and previous fractures (Tables 1, 2 and S1).

To determine the effect size of the risk factors, we used the cox regression for the survival risk analysis. We calculated a significantly higher risks with older age (*p* < 0.001), lower renal function (*p* = 0.010), lower handgrip (*p* < 0.001) and slower walking speed (*p* = 0.001). Significantly higher mortality was seen in men (*p* < 0.001), patients with lower FRAX score (p = 0.010), lower Tinetti test (*p* = 0.012) and lower Barthel index (*p* = 0.015) (Table 4). A further analysis using the logistic regression revealed a significant odds ratio for sarcopenia (*p* = 0.029) and lower DI values (*p* = 0.012) in addition to the findings in the cox regression analysis (Table 5).

**TABLE 5:** Logistic regression analysis of significant parameters for fractures (A) and death (B).

(A)					
Parameter	B	Odds ratio	Lower bound	Upper bound	*p*
Body mass index	−0.091	0.913	0.840	0.993	**0.034**
T‐value	−0.364	0.694	0.512	0.941	**0.019**
Osteoporosis	0.824	2.280	1.111	4.681	**0.025**
Osteopenia	−0.137	0.872	0.432	1.757	0.701
Osteosarcopenia	1.008	2.740	1.008	6.903	**0.032**
Parathyroid hormone	−0.018	0.982	0.964	1.000	**0.047**

(B)					
Parameter	B	Odds ratio	Lower bound	Upper bound	*p*
Age	0.083	1.086	1.023	1.153	**0.006**
Body mass index	−0.084	0.919	0.851	0.992	**0.033**
Glomerular filtration rate	−0.019	0.981	0.967	0.995	**0.008**
Sarcopenia	0.444	1.558	1.047	2.318	**0.029**
Handgrip	−0.069	0.933	0.884	0.985	**0.012**
Walking speed	−2.126	0.119	0.015	0.941	**0.044**
DI	−4.490	0.300	0.001	0.768	**0.037**
Low DI value	0.928	2.530	1.222	5.239	**0.012**

*Note:* Data with significant values are presented in bold.

**FIGURE 2:**
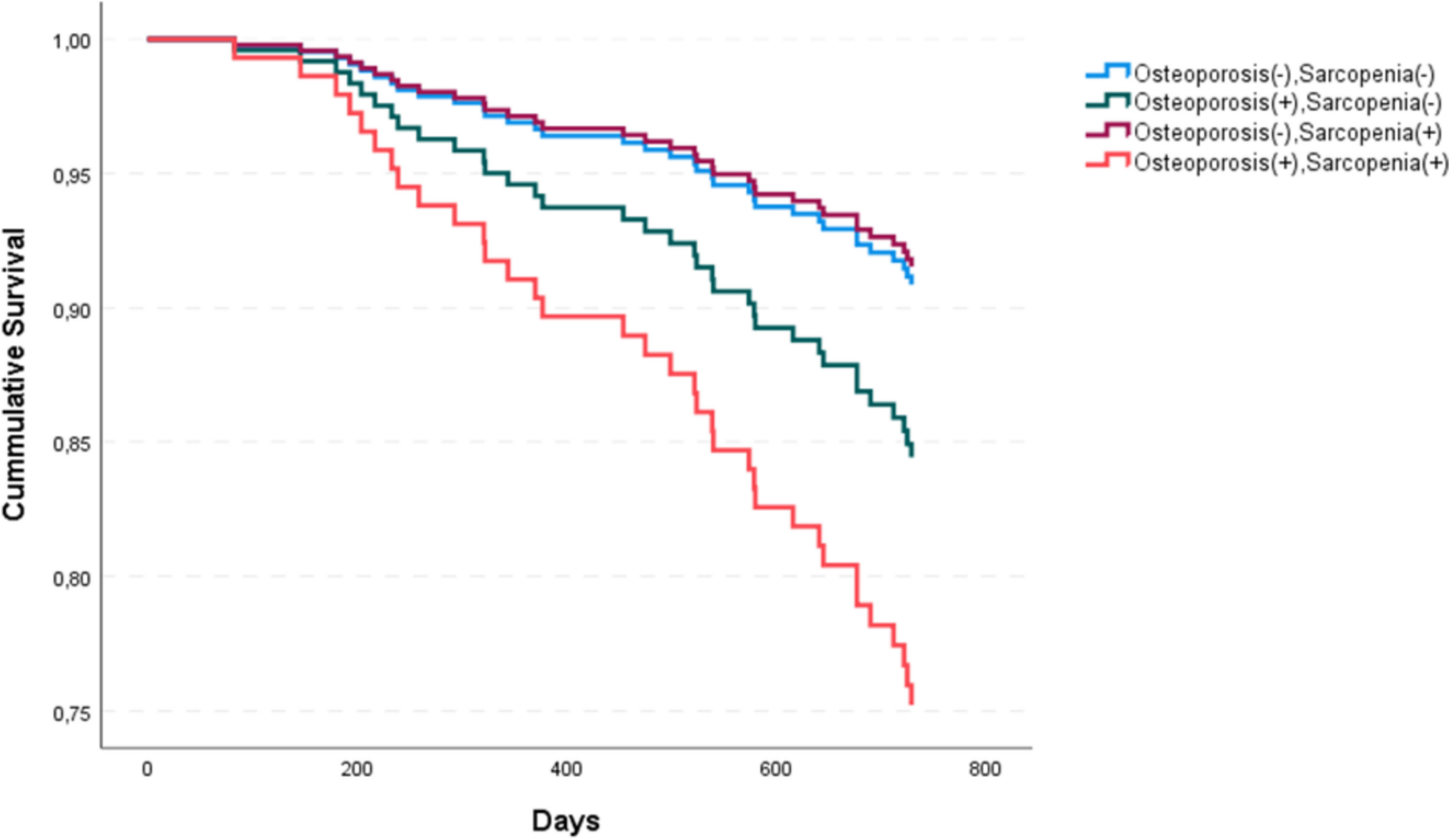
Kaplan Meier curve: time to fracture in osteoporosis, sarcopenia and osteosarcopenia or none.

**FIGURE 3:**
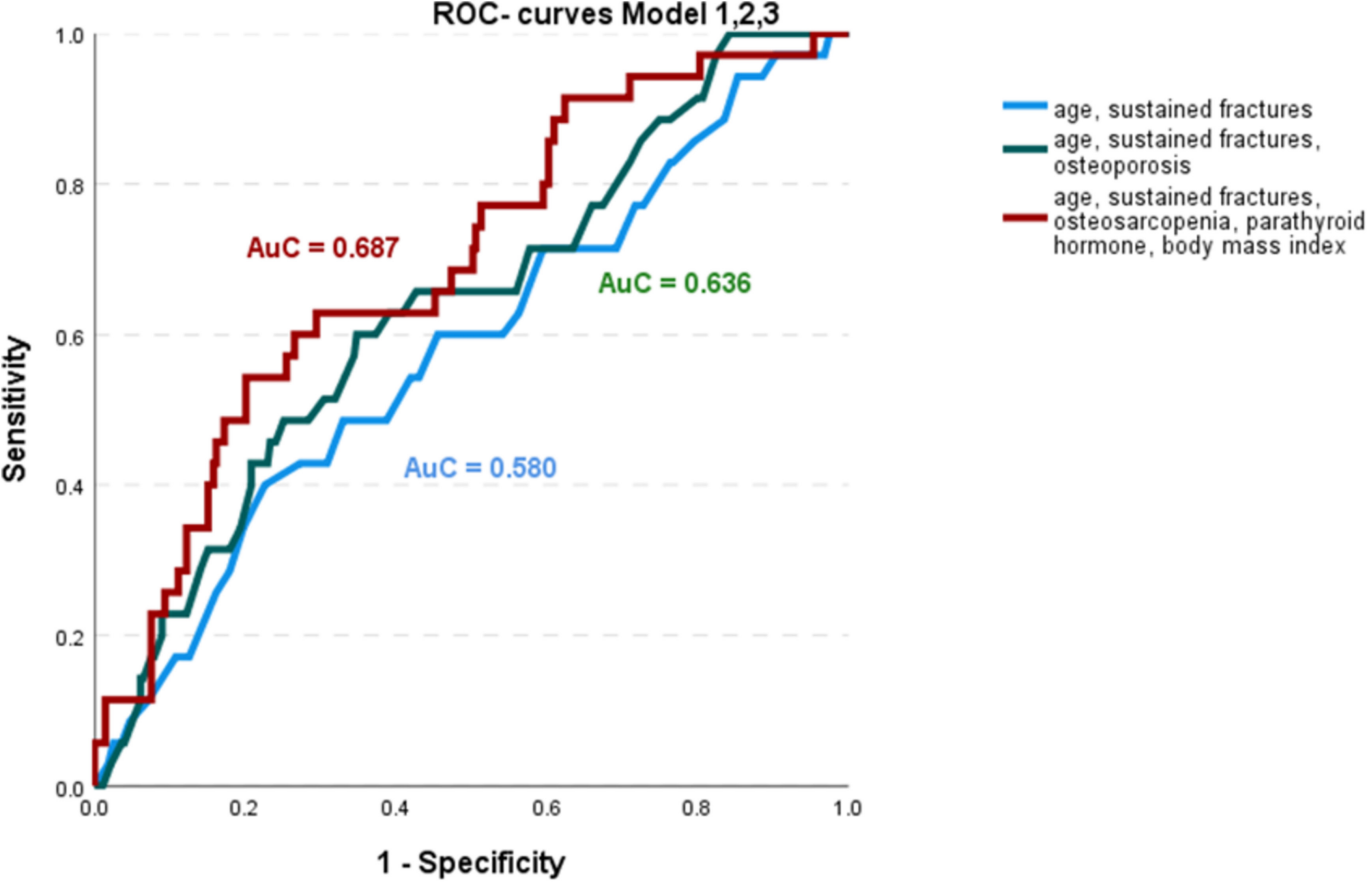
ROC curves of 3 models using the logistic regression analysis for further fractures within 24 months.

To visualize the effects on further bone fractures, we constructed a Kaplan–Meier curve plotting the time to fracture with parameters of bone fragility, sarcopenia and osteosarcopenia. This curve revealed a continuously increasing risk for fractures that separated distinctively after 200 days (Figure 2). We built 3 models for the prediction of further fractures based on data of the regression analysis over the period of 24 months (Figures 3 and S1). The ROC curves indicated a low predictive power of age and sustained fractures at baseline (model 1) with an area under the curve of 0.580. A higher value of 0.636 was seen adding osteoporosis (present or not) to the model (model 2), and an area under the curve of 0.687 adding osteosarcopenia (present or not), BMI and the parathyroid hormone level (model 3). The predictive power was 10.7% higher in model 3 versus model 1.

## 4 Discussion

This is an initial study in a specific cohort of geriatric patients that reveals the predictive power of parameters of muscle health, bone health and the functionality parameters for further fragility fractures and mortality within 2 years.

New fractures occurred in 7.9% (3/38) of cases with sarcopenia and in 16.3% (8/49) of osteoporotic patients in our study, but as many as 28% (7/25) patients with osteosarcopenia. This association of sarcopenia and fragility fractures was implied in a systematic review by Wong et al. using the previous definition of the European Working Group on sarcopenia in mainly younger patients proposing a hazard ratio of 2.986 (95% CI, 2.402–3.711) for further fractures. As important fact emerged, the different hazard rate for further fractures using ‘pure’ data on sarcopenia, osteoporosis, osteosarcopenia or none in comparison to data of patients without exact differentiation presented in previous studies [30]. In our study, we found odds ratios and hazard ratios in line with the published data analysing patients on sarcopenia or osteoporosis in general, but a significant association for further fractures only in women doing an exact separation of groups of patients with osteoporosis, sarcopenia, osteosarcopenia or none (Table 3).

Lower body mass was significantly associated with a higher rate of further fractures. This association corresponds with a general population‐based cohort study in Korea with hazard ratio of 1.165 (95% CI 1.113–1.218) [31]. Lower body mass, often associated with malnutrition, induces osteoporosis and bone degeneration. Furthermore, low body weight is associated with the development of sarcopenia followed by reduced muscle function and falls, increasing the risk of fractures.

Despite the well‐known high association of refracture in patients with previous fragility fractures, our cohort of patients did not show this association. Only 12.2% (24/197) of patients with fractures at baseline suffered a new fragility fracture within 2 years. That was 5.8% lower than among 377 561 female Medicare beneficiaries who sustained a fracture: 10% had another fracture within 1 year, 18% within 2 years and 31% within 5 years [1]. We hypothesized that the treatment of all patients along contemporary guidelines on osteoporosis at baseline influenced the refracture rate as presented by LeBoff et al. [32]. Our data go in line with an intervention study on 59 926 patients with osteoporosis that showed a reducing effect of 8%–11% in 3 years on specific treatment, despite the fact that our study was not designed for treatment analysis [33].

Nevertheless, the strongest predictor for refracture was osteoporosis, respectively, a low BMD. We calculated a hazard ratio of 2.546 (95% CI 1.192–5.438; *p* = 0.016) for a new fracture in the observational period of 24 months in patients with osteoporosis after baseline evaluation. Our intention to predict the refracture rate with pulse‐echo ultrasound measurement revealed no significant results in contrast to the traditional DXA measurements showing a hazard rate of 0.211 (95% CI 0.012–3.712; *p* = 0.288) for the prediction of new fracture in that period.

Osteosarcopenia, the novel geriatric syndrome, should be considered a geriatric giant of the XXI century due to its high prevalence in older persons and the vast personal burden of sufferers [17]. We found it significantly more prevalent in 7 cases (20%) in the group of patients with new fractures versus 25 cases (8.4%) in the group without. The significant odds ratio of 2.740 (*p* = 0.032) confirmed this association. The data fit well in a meta‐analysis of Huang et al. in 15 062 patients with a prevalence of 17%–24% depending on the definition of sarcopenia and the clinical setting [34]. We emphasize the need of an exact diagnosis and medical treatment of both clinical entities in geriatric patients with a clinical profile equivalent to our cohort to prevent further fragility fractures.

Assessments of frailty, walking speed, grip strength and skills of daily life did not predict further fractures in our study, despite previous published association of phenotypical frailty and following hip fracture (multivariate HR = 1.40, 95% CI, 1.03–1.90) and any non‐spine fracture (multivariate HR = 1.25, 95% CI, 1.05–1.49) in older women and men by Ensrud et al. [35]. We interpreted the difference in the longer follow‐up period of the previous published data.

The significantly higher rate of new fractures in correlation with low levels of parathyroid hormone in our study goes in line with the pathophysiologic effect of lower bone remodelling and lower bone quality in hypoparathyroidism, although the effect was small in our cohort with an odds ratio of 0.984 (95% CI 0.966–1.000; *p* = 0.047). These data are consistent with the reported increase in the risk of fractures (vertebral body fractures) in patients with hypoparathyroidism in the review by Pal et al., although the data on fracture risk are controversial [36]. In our study cohort, 70.9% of the patients had vitamin D insufficiency [9], which did not affect the risk of fractures (Table 1) but did not cause an increase in parathyroid hormone, as would be expected physiologically. One explanation can be found in the concept of functional hypoparathyroidism in geriatric patients with vitamin D deficiency and low parathyroid hormone levels, as shown in the study by Björkman et al. on 218 immobile patients [37].

Mortality calculations revealed significant associations of sarcopenia and death within 24 months with an OR of 1.558 (95% CI 1.047–2.318). That was in line with data of a meta‐analysis of Xu et al. and an OR of 2.351 (95% CI 1.638–3374) [38]. We can explain the difference by the various diagnostic method of assessing sarcopenia, using the old classification in the meta‐analysis of Xu et al in most of the cited studies and the new classification in our study. This difference is a well‐known consequence of applying the new diagnostic guidelines in older adults [39]. The even more skilled assessment of muscle function recording hand grip strength and walking speed (both inversely associated with sarcopenia), showed significantly inversed risk of mortality in our study. These easily recordable parameters can be integrated in the clinical routine offering significant data on prognosis and the appropriate dosing of further therapeutic measurements.

For the first time we could demonstrate an inverse association of low DI values, analysed with pulse echo ultrasound, with mortality. Despite of known data on higher mortality in patients with osteoporosis, we found no significant association in our study [40]. These contradictory findings can be resolved by the fact that the DI values represent measurement of the cortical thickness, combined with data regarding the patient's age, weight and height versus the classical diagnostic approach for osteoporosis using DXA measurements. Therefore, the low DI values went in line with the significant OR of age and low BMI in our study.

### 4.1 Limitations

A limitation represents the single‐centre conception of our study. The sample size was small and slightly lower than that reported in common studies. On the other hand, we calculated an extraordinary consistency of mortality and fracture risk values with international studies in other ethnicities relativizing this disadvantage. The high therapy adherence of our cohort in a local setting of the Salzkammergut area that has only one medical provider for inpatients obtains optimal requirements for this longitudinal follow up study. Secondly, our data presents the results of an ongoing follow up study in the first 24 months with small, but significant odds ratios compared with studies on data of long follow up studies. Geriatric patients, given the short lifespan left, need timely therapeutic approaches, so the period of 24 months in our study seems quite appropriate.

### 4.2 Strengths

We could clearly demonstrate along our presented models a gain of predictive power for further fractures and mortality within 24 months, using additional clinical findings from the functionality assessment in conjunction with the known risk factors osteoporosis and sarcopenia in a precisely defined group of geriatric patients. The different significance of data using the diagnosis of osteoporosis and sarcopenia in general versus the distinct analysis of the presented 4 groups of patients is remarkable.

### 4.3 Conclusions

We concluded that the addition of BMI, low parathyroid hormone levels and osteosarcopenia increases the predictive power for the risk of further fragility fractures in addition to advancing age and a history of sustained fractures. These risk factors are potentially treatable and lead to geriatric co‐management of geriatric trauma. Mortality was associated on a large scale with functionality parameters using the geriatric assessment, factors suggested as targets for therapeutic interventions.

## Ethics Statement

All authors affirmed to comply with the ethical guidelines of authorship published in the journal.

## Consent

Participants provided their informed approval on a declaration of consent form. We conducted the research on a consent of the local ethics committee in Upper Austria under the project title: ‘Pulse‐echo ultrasound measurement of bone mineral density as a detection device for osteoporosis in geriatric patients – a prospective exploratory study’ with the EK Nr: 1103/2018.

## Conflicts of Interest

The authors declare no conflicts of interest.

## Supporting information


**Data S1.** Supporting information

**Figure S1.** Supporting information

**Table S1.** Supporting information

**Data S2.** Supporting information
