# Supplementary material for: Prediction of Fragility Fractures and Mortality in a Cohort of Geriatric Patients
Source: J Cachexia Sarcopenia Muscle. 2024 Nov 8;15(6):2803–14. doi: 10.1002/jcsm.13631 (PMC11634494; doi:10.1002/jcsm.13631)
Supplement: Supplementary file 1 — Data S1. Supporting information [file JCSM-15-2803-s003.docx]

**I. Inclusion criteria**

1. Men and women with the clinical profile of a geriatric patient based on the definition of the German Geriatric society (Sieber, C. The older patient – who is it? Internist 48, 1190–1194 (2007): patients with an age above 70 years and multimorbidity, typical for a geriatric patient, or patients with an age of 80 years or older, and higher vulnerability based on the definition of frailty (Fried LP, Tangen CM, Walston J, Newman AB, Hirsch C, Gottdiener J, Seeman T, Tracy R, Kop WJ, Burke G, McBurnie MA; Cardiovascular Health Study Collaborative Research Group. Frailty in older adults: evidence for a phenotype. J Gerontol A Biol Sci Med Sci. 2001) and

2. A risk factor of osteoporosis due to the assessment of clinical risk factors used for the assessment of fracture probability (Compston, J., Bowring, C., Cooper et al. (2013). Diagnosis and management of osteoporosis in postmenopausal women and older men in the UK: National Osteoporosis Guideline Group (NOGG) update 2013. Maturitas, 75(4), 392–396. doi:10.1016/j.maturitas.2013.05.013):

Age 70 years

Female Sex

Low body mass index (≤19 kg/m2)

Previous fragility fracture, particularly of the hip, wrist and spine including morphometric vertebral fracture

Parental history of hip fracture

Current glucocorticoid treatment (any dose, by mouth for three months or more)

Current smoking

Alcohol intake of three or more units daily

Secondary causes of osteoporosis including:

Rheumatoid arthritis

Untreated hypogonadism in men and women

Prolonged immobility

Organ transplantation

Type I diabetes

Hyperthyroidism

Gastrointestinal disease

Chronic liver disease

Chronic obstructive pulmonary disease

Falls

**Exclusion criteria:**

1. Patients receiving ongoing treatment for osteoporosis

2. Patients without both lower extremities

3. Patients with an inflammatory bone disease

4. Patients with a neoplastic bone disease

5. Patients unable to provide written consent
