# Supplementary material for: Prediction of Fragility Fractures and Mortality in a Cohort of Geriatric Patients
Source: J Cachexia Sarcopenia Muscle. 2024 Nov 8;15(6):2803–14. doi: 10.1002/jcsm.13631 (PMC11634494; doi:10.1002/jcsm.13631)

Supplement figure ROC curves of 3 models using the logistic regression analysis for further fractures within 24 months


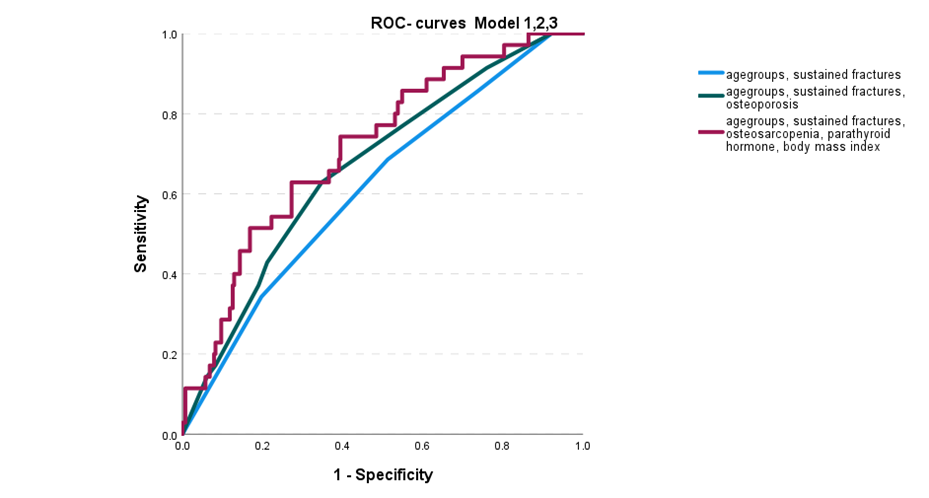

Supplement: Supplementary file 2 — Figure S1. Supporting information [file JCSM-15-2803-s004.docx]
