# Supplementary material for: Prediction of Fragility Fractures and Mortality in a Cohort of Geriatric Patients
Source: J Cachexia Sarcopenia Muscle. 2024 Nov 8;15(6):2803–14. doi: 10.1002/jcsm.13631 (PMC11634494; doi:10.1002/jcsm.13631)
Supplement: Supplementary file 3 — Table S1. Supporting information [file JCSM-15-2803-s001.docx]

**Supplement table** Subgroup analysis of the study population stratified by occurrence of new fractures and survival within 24 months

| **Characteristics** | **Total (334)** | **No further fracture (299)** | **New fracture (35)** | **p-value** | **Survived (293)** | **Deceased (41)** | **p-value** |
| --- | --- | --- | --- | --- | --- | --- | --- |
| **Age group < 80 years (%)** | 141 (42.2) | 123 (41.1) | 18 (51.4) | 0.164 | 128 (43.7) | 13 (31.7) | **0.004** |
| **Age group 80-89 years (%)** | 169 (50.6) | 152 (50.8) | 17 (48.6) |  | 149 (50.9) | 20 (48.8) |  |
| **Age group >90 (%)** | 24 (7.2) | 24 (8.0) | 0 (0) |  | 16 (5.5) | 8 (19.5) |  |
| **Diabetes mellitus type II** | 69 (20.7) | 62 (20.7) | 7 (20.0) | 0.919 | 235 (80.2) | 58 (19.8) | 0.297 |
| **Hyperthyroidism *** | 21 (6.3) | 19 (6.4) | 2 (5.7) | 0.435 | 17 (5.8) | 4 (9.8) | 0.360 |
| **Hypothyroidism *** | 53 (15.9) | 50 (16.8) | 3 (8.6) |  | 49 (16.8) | 4 (9.8) |  |
| **Malnutrition **** | 87 (26.1) | 80 (26.8) | 7 (20.0) | 0.383 | 69 (23.6) | 18 (43.9) | **0.006** |
| **Chronic obstructive respiratory disease** | 28 (8.4) | 24 (8.0) | 4 (11.4) | 0.492 | 20 (6.8) | 8 (19.5) | **0.006** |
| **Smoker (current)** | 25 (7.5) | 23 (7.7.) | 2 (5.7) | 0.670 | 17 (5.8) | 8 (19.5) | **0.002** |
| **patients with long-term corticoid medication §** | 7 (2.1) | 5 (1.7) | 2. (5.9) | 0.153 | 4 (1.4) | 4 (7.3) | **0.043** |

*The reference range defined by the 2.5th and 97.5th percentiles of the circulating thyroid hormone values in healthy population.**Patients with an unintended weight loss >5% within 3 months and a BMI < 19 were counted as malnourished.§ Patients on systemic corticoid therapy for longer than 6 weeks due to comorbidity.
