# Supplementary material for: Prediction of Fragility Fractures and Mortality in a Cohort of Geriatric Patients
Source: J Cachexia Sarcopenia Muscle. 2024 Nov 8;15(6):2803–14. doi: 10.1002/jcsm.13631 (PMC11634494; doi:10.1002/jcsm.13631)
Supplement: Supplementary file 4 — Data S2. Supporting information [file JCSM-15-2803-s002.docx]

**Trail protocol**

“Pulse-echo ultrasound measurement of bone density for the diagnosis of osteoporosis in geriatric patients”

Responsibilities and addresses:

Head of clinical trial: Peter Dovjak, MD, head of Acute Geriatrics, Salzkammergutklinikum Gmunden, Miller von Aichholzstraße 49 , 4810 Gmunden Telephone +437612 796 33500 Email peter.dovjak@gespag.at

Project collaboration: Prof. Dr. Peter Pietschmann, head of department at the Institute of Pathophysiology, institute of Pathophysiology at the Medical University of Vienna, Währinger Gürtel 18-20, 1090 Vienna, Telephone +431 404005126

PD DDr. Gregor Dovjak,specialist in radiology, Institution University Hospital for Radiology and Nuclear Medicine, Medical University of Vienna, Währinger Belt 18-20, 1090 Vienna

Test center: Department of Acute Geriatrics/Remobilization, Salzkammergutklinikum Gmunden, Miller von Aichholzstraße 49, 4810 Gmunden, Tel. +437612796 33500

Short version of the project:

Osteoporosis is a classic age- and gender-related disease. Despite advances in diagnostics, it remains underdiagnosed and continues to cause potentially preventable fractures, particularly in older patients. The reason is partly due to the dual absorptiometry examination (DEXA) measurement with a high access threshold for functionally impaired older patients, partly to underestimation of the fracture risk with the DEXA values and the fear of ionizing radiation during the examination. Pulse - echo ultrasound measurements of the bone offer a new technique to determine the cortical bone thickness, the bone density (BMD - bone mineral density) and the so-called bone density index (DI). Using a mobile handheld device from Bone Index Finland LTd., Kuopio, Finland, software v.2.0Drott - Bindex®, these values can be collected at the bedside or for outpatients in the office. The diagnosis of osteoporosis is made using the international classification 1Sarcopenia and osteoporosis are common entities in the elderly and cause a number of health problems 2. The term osteosarcopenia goes hand in hand and is referred to as a dangerous duo for falls and fractures. Both conditions, if diagnosed in a timely manner, can be treated and the dangerous duo can be avoided. The diagnosis of sarcopenia is easily determined using a diagnostic algorithm from a European consensus conference 3.

Although studies comparing pulse-echo ultrasound values in a pilot study correlated well with DEXA values, no data are known for geriatric patients 4,5. No knowledge exists whether the data collected from the pulse-echo ultrasound examination predicts the risk of fracture over the long term. Therefore, this study asks these questions and sheds light on them based on the data collected.

Study participants:

Study participants with a geriatric profile according to the international definition who are treated as inpatients or outpatients at the Gmunden Acute Geriatric Clinic are included. Patients who cannot give consent will be excluded. Patients undergoing specific osteoporosis therapy and other bone diseases other than osteoporosis are also excluded.

Hypothesis:

1. In geriatric women and men, the further risk of fracture can be predicted using pulse echo ultrasound examination.

2. The bone density parameters measured with the ultrasound device are not inferior to the predictive values for osteoporosis of the DEXA measurement in geriatric patients.

Study objective:

Gender-specific differences in examination methods and long-term courses are recorded. The correlations with the pulse-echo ultrasound data and DEXA measurements are shown for geriatric patients as well as using the Altman Bland plot. The correlation with the risk assessments of the FRAX method and the pulse-echo ultrasound data are collected. The data collected will be examined separately for patients with fragility fractures and the control group.

Method:

All patients undergo a medical history, status assessment and geriatric assessment as part of clinical routine. Laboratory tests, DEXA measurements of bone density and muscle mass are carried out in accordance with the guidelines for the examination of osteoporosis and the diagnostic criteria for sarcopenia. In addition, ultrasound measurements are carried out on the extremities to determine bone quality parameters and the FRAX score is recorded. For follow-up examinations, patients are examined during subsequent stays in acute geriatrics or during an outpatient check-up. If this is not possible, a telephone interview will be carried out with the patient or, in the case of functional limitations, with the nursing staff.

Laboratory parameters collected:

Blood count, calcium, phosphorus, alkaline phosphatase, alanine transferase, aspartate transferase, gamma glutamyl transferase, nitrogen, creatinine, albumin, total protein, erythrocyte sedimentation rate, electrophoresis, thyroid stimulating hormone. For possible further investigations into the pathophysiology of bone metabolism, the serum of 8 ml of blood is stored at -70°.

Ethical and legal aspects:

The study will be conducted in accordance with the guidelines of the Declaration of Helsinki (1964, including later editions). All study participants sign a consent form after receiving detailed information about the background, aim, possible consequences and benefits of the study. The study protocol will be submitted to the ethics committee in Upper Austria for approval before the study begins.

Table of contents

1.Scientific background

2.Study objective

2.1.Design

2.2.Target parameters

3.Recruitment

3.1. Number of subjects and duration of the study

3.2.Selection of test subjects

3.2.1.Inclusion criteria

3.2.2.Exclusion criteria

3.3.Investigation

3.3.1.Anamnese

3.3.2.Physical status

3.3.3.geriatric assessment

3.3.4.Laboratory parameters

3.3.5.DEXA measurement

3.3.6.Ultrasound measurement

4.Treatment of study participants

4.1.Inclusion in the study

4.2.Location of the study

4.3.Nutrition

4.4.Concomitant therapy

4.4.1.permitted concomitant therapy

4.4.2. inadmissible concomitant therapy

4.5.Documentation

5. Study procedure: description, scheme, duration, running time, schedule, handling of pre-medication, examinations

5.1.Methods

5.1.1.Transportation

5.1.2.Preparation of serum samples

5.1.3.Laboratory analysis

5.1.4.Validation

5.1.5.Reliability

5.1.6.Compliance recording

5.2.DEXA measurement

5.3.Pulse-echo ultrasound measurement

5.4.Risk-benefit assessment

5.5.Adverse Events

5.6. Termination of the study

6.Biometrics

7.Change to the test plan

8.Legal basis

9.Investigator

10.Archiving, data protection

11.Publication

12.Signatures

13.Literature

Attachment

I. Patient information and declaration of consent

II. Test sheet

1.Scientific background

According to the consensus definition from 2001, osteoporosis is a generalized disease of the skeletal system that leads to an increased risk of fractures due to a disruption in bone strength 1. Nevertheless, the treatment of osteoporosis is suboptimal in high-risk patients and even after a fracture 6. One in two women will suffer an osteoporotic fracture in their lifetime, with a mortality rate higher than the combined mortality rate from breast and ovarian cancer. Osteoporotic fractures near the hip cause pain, loss of function, increase the risk of further injury from falls, long hospital stay and rehabilitation 7. The pathophysiology of hip fractures in old age points to two factors - bone strength and the energy that led to the trauma. In people over 65 years of age, 90% of fractures result from a fall from standing or from a low height and therefore correspond to an osteoporotic fracture 8. An obligatory medical task of acute geriatrics is, in addition to pain control, thrombosis prophylaxis, coordination of remobilization, treatment of complications and underlying medical diseases, in patients after a fracture near the hip or with identified risk factors, the diagnosis and treatment of the seemingly always underlying osteoporosis in patients with a fracture near the hip 9. According to this task, the detection of osteoporosis and treatable forms of osteoporosis is medically indicated 10. The geriatric assessment is mandatory upon admission to acute geriatrics. The data to date indicate a clinical benefit for patients with falls, femoral neck fractures and a number of other geriatric syndromes when the appropriate tests are carried out and the changes found are treated 11. Ultrasound examinations to assess bone quantity and quality have been used in clinical practice for more than three decades 12. The combination of a new ultrasound technique - the pulse-echo examination with DEXA has proven to be helpful in the case of borderline findings in DEXA4. The examination can be used as a screening for osteoporosis 5. The International Osteoporosis Foundation and guidelines recommend the use of the FRAX® method to determine fracture risk and osteoporosis screening 1. Secondary fall prevention in patients after falls, fractures or increased risk of falls by means of fall analysis and treatment of the identified medical causes results in a 20-40% reduction in further falls 13. The detection and treatment of sarcopenia in particular has proven to be additionally effective 14-16.

2.Study objective, hypothesis

This prospective study aims to demonstrate the predictive value of pulse-echo ultrasound for fractures.

Gender-specific differences in examination methods and long-term courses are recorded. The correlation with the pulse – echo ultrasound data and DEXA measurements are presented for geriatric patients. The correlation with the risk assessments of the FRAX method and the pulse-echo ultrasound data are collected. The data collected will be examined separately for patients with fragility fractures and the control group.

2.1.Design

Prospective cohort analysis

2.2.Target parameters

History, clinical status and the geriatric assessment.

Blood count, calcium, phosphorus, alkaline phosphatase, alanine transferase, aspartate transferase, gamma glutamyl transferase, nitrogen, creatinine, albumin, total protein, erythrocyte sedimentation rate, electrophoresis and thyroid stimulating hormone.

For possible further scientific investigation of parameters of bone metabolism or of hormones, cytokines or other factors that influence bone metabolism, the serum of 8ml of blood is stored at -70°Celsius.

DEXA measurement with measurement of muscle and bone mass: The skeletal muscle index (muscle mass of the arms and legs divided by the square of the height in meters) and the T-value (deviations from the age- and gender-specific normal in multiples of a standard deviation) are recorded. . In addition, ultrasound measurements are carried out on the extremities to determine global bone density and cortical thickness (in mm) and the FRAX score (evaluated for Austrian patients) is collected.

3.Recruitment

All geriatric patients who are admitted to the department for acute geriatrics/remobilization at the Salzkammergutklinikum Gmunden or who are treated as outpatients with an increased risk of osteoporosis and who are treated during the same period will be included in the study after information and their consent.

3.1. Number of subjects and duration of the study

Due to the exploratory nature of the study and the lack of data for this collective, the sample size is determined based on the following considerations: The overall fracture rate for all people over 50 years of age is 3% in 17. The fracture rate in osteoporosis is increased depending on bone density and is related to the pulse rate. Echo ultrasound machine with a sensitivity of 89% predicted 4,5,18. With a bone density with a T value of -2.5, the fracture rate is approximately tripled and is therefore 9%. Therefore, the following sample size calculation results with an α error of 0.05 and a power (1-β) of 0.8 with patients without osteoporosis and a normal fracture risk of 3% annually divided by 9% for patients with osteoporosis. A number of cases of 161 are required to detect clinically relevant osteoporosis and a number of 1491 cases are required to detect osteopenia.

3.2.Selection of test subjects

At the start of the studies, study participants are consecutively selected by the head of the clinical trial at the Department of Acute Geriatrics/Remobilization in accordance with the inclusion criteria for study participation and for the group of control subjects.

3.2.1.Inclusion criteria

Geriatric men and women with a risk factor for osteoporosis. Patients at high risk of osteoporosis are identified according to the history and status and the guidelines for the diagnosis and management of osteoporosis according to J.Compston et al. recognized 19.

3.2.2.Exclusion criteria

• Patients who do not sign the informed consent form.

• Patients with specific osteoporosis therapy.

• Patients with a bone disease other than osteoporosis.

3.3.Investigation

As part of the routine procedure for admission to acute geriatrics, the anamnesis, clinical status, geriatric assessment and, depending on the risk constellation, further examinations to clarify osteoporosis are collected.

3.3.1.Anamnese

As part of clinical routine, the anamnese is taken upon admission to the acute geriatrics/remobilization department and the relevant parameters for the study are transferred from the data to the test form.

3.3.2.Physical status

As part of clinical routine, the physical status is recorded upon admission to the acute geriatrics/remobilization department and the relevant parameters for the study are transferred from the data to the test form.

3.3.3.Geriatric assessment

The assessment is a multidimensional diagnostic procedure for geriatric patients to examine their medical, psychological and functional abilities in order to develop a coordinated treatment plan. It contains a catalog of tests recommended for acute geriatrics by the Society for Geriatrics and Gerontology, which are carried out within 72 hours in an interdisciplinary procedure after admission to the acute geriatrics/remobilization department. In particular, cognition is assessed using the clock test according to Shulman14 and the Mini Mental State Examination according to Folstein15, for functionality the Barthel Index 16, for nutritional status the Mini Nutrition Assessment17 and for testing mobility the Tinetti Test18 and the Timed Get-Up and Go Test19 used. To diagnose sarcopenia, gait speed and hand strength are measured

3.3.4.Laboratory parameters

As part of clinical routine, upon admission to the acute geriatrics/remobilization department, a fasting blood sample is taken with analysis of the blood count, serum parameters of kidney function, liver function, electrolytes and inflammatory parameters. Patients with osteoporosis are treated in accordance with the guidelines.20, 21

3.3.5.DEXA measurement

As part of the clinical routine for the diagnosis of sarcopenia and/or osteoporosis, patients are examined in the radiological institute of the Salzkammergutklinikum Gmunden after informed consent and a declaration of consent in accordance with clinical practice. The Lunar device from General Electrics, Madison, WI, USA is used for the standardized examination of bone density and muscle mass determination in routine clinical operations as part of the clinical safety standards for patients.

3.3.6.Pulse – echo ultrasound measurement

Using the commercially available hand-held device Bindex from Bone Index Finland Ltd., Kuopio, Finland, pulsed ultrasound waves with a frequency of 3 megaherz are applied to the lower leg of the study participants by the trained examiner and the measured values collected by the device are transferred to a connected commercially available laptop and read. The examination lasts on average 30 seconds and is painless.

4.Treatment of study participants

The study participants are treated as part of clinical routine at the Department of Acute Geriatrics/Remobilization in accordance with the therapy plan drawn up and under the responsibility of the specialist in charge of the bed.

Study participants must be withdrawn from the study at their own request if they violate the study plan. In these cases, detailed documentation is provided.

4.1.Inclusion in the study

Study participants who have signed the informed consent form and meet the inclusion criteria will be included in the study.

4.2.Location of the study

Department of acute geriatrics/remobilization at the Salzkammergutklinikum Gmunden

4.3.Nutrition

The study participants have no study-specific restrictions

4.4.Concomitant therapy

The medication and physical treatments during the stay in the acute geriatric ward/remobilization are carried out by the responsible internal medicine specialist in accordance with the therapy plan that is independent of study participation.

4.4.1.permitted concomitant therapy

The medication is administered as prescribed by the responsible physician during the inpatient stay in the acute geriatric ward/remobilization, regardless of participation in the study.

4.4.2. inadmissible concomitant therapy

No study-specific medication restrictions are planned.

4.5.Documentation

All data is recorded on the test sheet (see appendix) for further analysis.

The data will be stored securely by the investigator. The data is used pseudo-anonymously for further investigation.

5.Study procedure

After approval by the ethics committee, the study will be conducted for 12 months. According to the estimate (point 3.1.), 200 patients will be included. After giving their consent, the study participants undergo routine clinical treatment and the relevant data are collected from the anamnesis, clinical examination and blood test. Blood is drawn from the vein on an empty stomach after at least 12 hours of fasting. The DEXA measurement and ultrasound measurement within 7 days of admission.

5.1.Methods

5.1.1.

The blood sample is taken on normal working days of the week between 7:00 a.m. and 8:00 a.m. while sitting or lying down under resting conditions. After applying the tourniquet and disinfecting the skin with Isocid-H colorless from Gebro, Austria, a superficial vein in the elbow region is punctured. After loosening the tourniquet hose, the Vacuettes from Greiner Austria are filled with gentle suction up to the mark. 5 tubes are required per patient. One tube for the erythrocyte sedimentation rate coated with trisodium citrate 4:1 with a volume of 1.6 ml, one tube for the blood count examination with EDTA dipotassium and EDTA tripotassium salt coated with a volume of 2 ml, 3 tubes for the serum tests with coagulation activator and granules coated with 4 ml each. A total of 15.6 ml of blood is taken for the routine examination during inpatient admission. After the removal has been completed, the puncture needle is removed and the puncture site is treated with cotton-cellulose swabs from Hartmann, Germany and Leukosilk plaster strips from Beiersdorf AG, Germany. The DEXA measurements and ultrasound measurements are carried out in accordance with points 3.4.5 and 3.4.6.

5.1.2.Transportation:

The filled blood tubes are further processed and analyzed within 3 hours in the central laboratory of the Salzkammergutklinikum Gmunden. For shipping, the centrifuged serum samples are packaged and transported by post. A centrifuged serum sample is frozen at -70° Celsius for further examinations within 3 hours.

5.2.2.Preparation of serum samples

After coagulation, the tubes for the serum analyzes are centrifuged for 10 minutes at 4000 revolutions per minute using the Rotixa 50 RS centrifuge from Hettich, built in 2001.

5.1.3.Laboratory analysis

The serum supernatant is pipetted and analyzed in an automatic process for processing in the Cobas 6000 from Roche. The analysis values for calcium, phosphate, creatinine in serum, alkaline phosphatase, alanine transferase, aspartate transferase, gamma glutamyl transferase, nitrogen in serum, thyroid stimulating hormone are created automatically and processed electronically.

The blood count test is carried out from the blood count tube in a fully automated process in the Sysmex XT-2000i, built in 2007. The values are processed electronically. The erythrocyte sedimentation rate measurement is carried out after pre-treatment of the tube with the Vacuette Mixer from Greiner, built in 2009, at 10 revolutions per minute for 2 minutes using the Westergren method in the Screener 100 from Greiner, built in 2010, and electronic reading and further processing of the values. The serum values for albumin are packaged after processing in the centrifuge and sent to the Salzkammergutklinikum Vöcklabruck, 20 km away, for analysis with the Dimension analyzer from Date/Behring and analyzed within 12 hours of collection.

5.1.4.Validation

The quality control of the samples is carried out internally daily using reference sera from Roche and three times a year by the round tests of the Austrian Society for Quality Assurance and Standardization of Medical Diagnostic Examinations in 1090 Vienna, Hörlgasse 18.

5.1.5.Reliability of the analysis:

The sample tubes are analyzed in the central laboratory of the Salzkammergutklinikum Gmunden and Vöcklabruck as part of routine operations. The blood test data is archived electronically on a patient-related basis for years.

5.1.6.Compliance recording

The compliance of the individual examinations is recorded in the test form.

5.2. DEXA measurement – see point 3.3.5.

5.3. Pulse echo ultrasonic measurement – see point 3.3.6.

5.4.Risk-benefit assessment

The risk for the study participants corresponds to blood collection as part of clinical routine. They benefit from the careful differential diagnostic clarification of bone metabolism and muscle status with the resulting medical treatment.

5.4.Risk-benefit assessment

The risk for the study participants corresponds to blood collection as part of clinical routine. They benefit from the careful differential diagnostic clarification of bone metabolism and muscle status with the resulting medical treatment.

5.5.Adverse Events

Adverse events during the study are intercurrent illnesses or accidents that may affect the well-being of the study participants and also alter laboratory values. The term adverse event is not causally related to the study. All adverse events will be followed until normalization or until the condition or death is adequately explained. In this case, an autopsy report is sought. All findings are documented in the test form under “adverse events”.

5.6. Termination of the study

In the event of new scientific aspects or clinical knowledge that clarifies the study's hypothesis, the study will be stopped early.

6.Biometrics

An Altman Bland analysis is carried out to demonstrate the comparability of both methods (DEXA and ultrasound measurement). To prove the correlations, the Pearson (r) and Spearman (rs) correlation coefficients are calculated. The X2 contingency table test was used to compare the frequencies. The Kruskal-Wallis test or ANOVA (for normally distributed data) is used to compare discontinuous variables.

7.Change to the test plan

If changes to the test plan are necessary or desired, they will be documented in writing. You need to report this to the ethics committee and to all authors.

8.Legal basis

The study will be conducted in accordance with the guidelines of the Declaration of Helsinki (1964) and subsequent revisions.

9.Investigator

Peter Dovjak MD, Head of the Acute Geriatrics/Remobilization Department, Salzkammergutklinikum Gmunden

10.Archiving, data protection

All data is recorded in the test form and stored securely by the investigator. The people involved are subject to confidentiality. Patients are assigned an identification number. The study participants will be informed about the data protection measures and patient protection measures. The data is passed on exclusively for statistical purposes; Without exception, patients are not mentioned by name. Patients will not be named by name in any publications of the data from this clinical study.

11.Publication

The results will be published by the study authors in scientific journals and medical conferences. The manuscript will be submitted to all authors for approval before publication.

12.Signatures

Peter Dovjak MD. Prof. Dr. Peter Pietschmann

Clinical investigator project member

Salzkammergutklinikum Gmunden Institute for Pathophysiology, MUW

References

1. Kanis JA, McCloskey EV, Johansson H, et al. European guidance for the diagnosis and management of osteoporosis in postmenopausal women. Osteoporos Int. 2013;24(1):23-57.

2. Drey M, Sieber CC, Bertsch T, Bauer JM, Schmidmaier R, group Fi. Osteosarcopenia is more than sarcopenia and osteopenia alone. Aging Clin Exp Res. 2016;28(5):895-899.

3. Cruz-Jentoft AJ, Landi F, Schneider SM, et al. Prevalence of and interventions for sarcopenia in ageing adults: a systematic review. Report of the International Sarcopenia Initiative (EWGSOP and IWGS). Age Ageing. 2014;43(6):748-759.

4. Karjalainen JP, Riekkinen O, Kröger H. Pulse-echo ultrasound method for detection of post-menopausal women with osteoporotic BMD. Osteoporos Int. 2018.

5. Schousboe JT, Riekkinen O, Karjalainen J. Prediction of hip osteoporosis by DXA using a novel pulse-echo ultrasound device. Osteoporos Int. 2017;28(1):85-93.

6. Solomon DH, Morris C, Cheng H, et al. Medication use patterns for osteoporosis: an assessment of guidelines, treatment rates, and quality improvement interventions. Mayo Clin Proc. 2005;80(2):194-202.

7. de Leur K, Vroemen JP, Vos DI, Elmans L, van der Laan L. Outcome after osteosynthesis of hip fractures in nonagenarians. Clin Interv Aging. 2014;9:41-49.

8. Zuckerman JD. Hip fracture. N Engl J Med. 1996;334(23):1519-1525.

9. Wang H, Li C, Zhang Y, et al. The influence of inpatient comprehensive geriatric care on elderly patients with hip fractures: a meta-analysis of randomized controlled trials. Int J Clin Exp Med. 2015;8(11):19815-19830.

10. Raisz LG. Clinical practice. Screening for osteoporosis. N Engl J Med. 2005;353(2):164-171.

11. Van Craen K, Braes T, Wellens N, et al. The effectiveness of inpatient geriatric evaluation and management units: a systematic review and meta-analysis. J Am Geriatr Soc. 2010;58(1):83-92.

12. Raum K, Grimal Q, Varga P, Barkmann R, Glüer CC, Laugier P. Ultrasound to assess bone quality. Curr Osteoporos Rep. 2014;12(2):154-162.

13. Tinetti ME, Baker DI, McAvay G, et al. A multifactorial intervention to reduce the risk of falling among elderly people living in the community. N Engl J Med. 1994;331(13):821-827.

14. Reginster JY, Beaudart C, Buckinx F, Bruyère O. Osteoporosis and sarcopenia: two diseases or one? Curr Opin Clin Nutr Metab Care. 2016;19(1):31-36.

15. Reginster JY, Cooper C, Rizzoli R, et al. Recommendations for the conduct of clinical trials for drugs to treat or prevent sarcopenia. Aging Clin Exp Res. 2016;28(1):47-58.

16. Bauer JM, Verlaan S, Bautmans I, et al. Effects of a Vitamin D and Leucine-Enriched Whey Protein Nutritional Supplement on Measures of Sarcopenia in Older Adults, the PROVIDE Study: A Randomized, Double-Blind, Placebo-Controlled Trial. J Am Med Dir Assoc. 2015.

17. Johnell O, Kanis JA. An estimate of the worldwide prevalence and disability associated with osteoporotic fractures. Osteoporos Int. 2006;17(12):1726-1733.

18. Siris ES, Miller PD, Barrett-Connor E, et al. Identification and fracture outcomes of undiagnosed low bone mineral density in postmenopausal women: results from the National Osteoporosis Risk Assessment. JAMA. 2001;286(22):2815-2822.

19. Compston J, Bowring C, Cooper A, et al. Diagnosis and management of osteoporosis in postmenopausal women and older men in the UK: National Osteoporosis Guideline Group (NOGG) update 2013. Maturitas. 2013;75(4):392-396.
